# Efficacy of Deep Dry Needling versus Percutaneous Electrolysis in Ultrasound-Guided Treatment of Active Myofascial Trigger Points of the Levator Scapulae in Short-Term: A Randomized Controlled Trial

**DOI:** 10.3390/life13040939

**Published:** 2023-04-03

**Authors:** Ana Isabel Benito-de-Pedro, Ricardo Becerro-de-Bengoa-Vallejo, Marta Elena Losa-Iglesias, David Rodríguez-Sanz, César Calvo-Lobo, María Benito-de-Pedro

**Affiliations:** 1 Facultad de Enfermería, Fisioterapia y Podología, Universidad Complutense de Madrid, 28040 Madrid, Spain; 2 Faculty of Health Sciences, Universidad Rey Juan Carlos, 28933 Alcorcón, Spain; 3 Facultad HM Hospitales de Ciencias de la Salud Universidad Camilo José Cela, 28692 Madrid, Spain

**Keywords:** clinical trial, dry needling, electrolysis, levator scapulae, neck pain

## Abstract

Deep dry needling (DDN) and percutaneous electrolysis (PE) provide the benefit of the mechanical effect of the needle, and PE adds the potential advantages of the galvanic current it incorporates in myofascial trigger points (MTrPs) therapy. The aim of this study was to compare the short-term efficacy between PE and DDN on active MTrPs of the levator scapulae by considering pain intensity. A simple-blind randomized controlled trial was carried out, recruiting patients suffering from non-specific neck pain lasting more than 3 months and with active MTrPs in the levator scapulae muscle (n = 52). Patients were divided into intervention (PE; n = 26) and control (DDN; n = 26) groups and received one treatment session on the active MTrPs of the levator scapulae. Patients were assessed for pain intensity, pressure pain threshold (PPT), cervical range of motion (CROM), neck disability and post-needling soreness, immediately after treatment, at 72 h and at 14 days. In addition, pain during treatment was recorded after the procedure. There were no significant differences for pain intensity, post-needling soreness and PPT. We found significant differences in CROM, immediately after treatment (*p* = 0.043), and at 72 h (*p* = 0.045), in favor of the PE group. Significant differences were found for neck disability (*p* < 0.047), immediately post-treatment, in favor of the DDN group. Moreover, there were significant differences for pain during the intervention (*p* < 0.002), in favor of the DDN group (4.54 ± 2.21) versus the PE group (6.54 ± 2.27). PE and DDN appear to have similar short-term effects. PE proved to be a more painful treatment than DDN. Clinical trial registry: NCT04157426.

## 1. Introduction

Non-specific chronic neck pain (NCNP), also called mechanical pain, is defined as neck pain with or without irradiation whose origin is unknown. In NCNP, there is no underlying structural pathology and it lasts more than 3 months [1,2,3]. It is estimated that in Western countries, half of the population will suffer from this disorder at some point in their lives [4]. Furthermore, according to data from the 2020 European Health Survey in Spain [5], NCNP is among the main chronic health problems suffered by the population over 15 years of age (11.33%).

To date, the pathophysiology is still not clearly known. However, in recent years, different studies have linked this condition to myofascial pain syndrome (MPS), establishing the presence of myofascial trigger points (MTrPs) as a relevant clinical sign in subjects with cervical spine disorders [6,7,8]. Thus, a study by Cerezo-Téllez et al. [6] found a 100% prevalence of MTrPs in patients with NCNP.

Non-invasive methods such as ultrasound or manual therapy [9] have been described for MTrP therapy. Minimally invasive methods such as deep dry needling (DDN), have also been described and DDN appears as one of the most common techniques in the treatment of NCNP [10]. DDN consists of the introduction of a filiform needle into the MTrP that reacts by producing a local twitch response (LTR) [10]. However, these treatments have continued to develop, leading to the emergence of percutaneous electrolysis (PE). PE involves passing a low-intensity galvanic current through a needle to generate a controlled local inflammatory response in the target tissue and thus promote a process of phagocytosis and subsequent regeneration of the damaged tissue [11,12].

The efficacy of DDN in relation to pain reduction has been widely demonstrated, especially in relation to immediate effects, comparing it with other types of manual therapy, sham or no treatment [13,14]. There is only low-level evidence that LTRs are necessary during treatment for greater efficacy; the issue is unclear and requires further investigation, as shown by Fernandez de las Peñas et al. [15].

PE has been attributed to advantages over DDN such as producing a greater number of LTRs, with fewer punctures, in addition to generating post-needling soreness and greater regenerative effects [16]. PE has been successfully used in MTrPs of muscles such as lateral ptrigoid [17], foot and leg musculature related to plantar heel pain [18] or the levator scapulae itself [19]. However, to our knowledge, there is no research on NCNP that compares and can confirm any additional benefit of PE versus DDN. It seems reasonable to think that patients can be improved by the mechanical effect of the needle and, in addition, the possible beneficial effects of the galvanic current can be added. We found several publications [6,8,20,21,22] in which levator scapulae appears as one of the main muscles contributing to NCNP. In most of them, treatment is performed on the trapezius. Hence, we chose levator scapulae as the target muscle in order to shed more light on its importance in such pathology. Therefore, the aim of this study was to compare the efficacy of DDN versus PE in reducing the painful symptomatology of patients suffering from NCNP caused by active MTrPs of the levator scapulae muscle.

## 2. Material and Methods

### 2.1. Design

A randomized, controlled and single blinded clinical trial was carried out between 28 September 2021, and 1 January 2022, involving two groups. This study was prospectively registered at ClinicalTrials.gov (accessed on 8 November 2019; identifier: NCT04157426) and approved by the ethics committee of the Hospital Clínico San Carlos (C.P.-C.I.13/021) following the regulations of the declaration of Helsinki. No funding was received to avoid conflict of interest.

### 2.2. Participants

Patients with NCNP were asked to participate in the trial. All of them were recruited from the Physiotherapy and Podiatry clinic FISIOFUENLA, located at C/Francia 34 bajo A (Fuenlabrada) 28943 Madrid, Registration No. CS5583, of the Community of Madrid, and gave written informed consent before participating in the study.

From the 62 patients recruited and who agreed to participate, 54 were finally selected as they met all the eligibility criteria. The first requirement was to have both active MTrPs in one of the levator scapulae muscles, and the principal investigator was responsible for identifying them by clinical examination as determined by Travell and Simons [23]. In spite of both active and latent MTrPs may evoke referred pain, active MTrPs were mainly differentiated secondary to the patients’ pain recognition according to the Delphi international consensus by Fernández-de-las-Peñas and Dommerholt [24]. All participants were evaluated by the same investigator who has extensive experience in the diagnosis and treatment of MPS, which increased confidence in the identification of MTrPs [25]. In addition, a diagnosis of neck pain for at least 3 months and being between 18 and 70 years of age were also inclusion criteria. Exclusion criteria included previous cervical injury; pregnancy; patients with cognitive impairment or any systemic disease; fibromyalgia; physiotherapy treatment in the study area during the last 4 weeks; contraindications to deep needling, needle apprehension or phobia; patients who were unable to speak, write or understand Spanish correctly.

### 2.3. Simple Size Calculation

Sample size was calculated with the G*Power 3.1.4.9.4 software (G*Power©; University of Kiel, Kiel, Germany) and was obtained according to the difference between two independent groups. The differences between the two independent means were sought using the independent Student’s *t*-test for a normal distribution with a two-tailed test, with an α level of 0.05, a 95% confidence interval, a desired statistical analysis power (1 − β) of 80% (error β = 20%) and an effect size of size of 0.8. Therefore, 26 participants were required in each group.

### 2.4. Study Variables

In the pretreatment assessment, first the principal investigator was in charge of the physical examination and of marking the two active MTrPs. Then, a second investigator, who remained blind to group assignment, was in charge of data collection (age, sex, height, weight, physical activity, occupation) and baseline assessment: pain intensity, pain after puncture, neck disability, pain threshold to pressure (PPT) and cervical range of motion (CROM). Immediately afterwards, the principal investigator was in charge of performing the interventions, DDN or PE, as well as recording any adverse effects that might result from the treatments. The secondary investigator then remeasured the same variables, adding the recording of perceived pain during treatment. Similarly, at 72 h and 14 days, pain intensity, post-needling soreness, neck disability, PPT and CROM were recorded again. Participants were instructed not to disclose their group assignment. 

#### 2.4.1. Pain Intensity

The participants reported their current pain intensity using the modified visual numeric pain scale (VNPS) adapted to Spanish. This is a version of the visual analogue scale (VAS), developed by González, VM. et al. [26], to which they added visual signals in the form of histograms that correspond in size and color to the numerical values, and in which patients must make a mark, scoring from 0 to 10, where higher values indicate more pain. The test–retest reliability of the VPNS is 0.64. VAS scale has good validity and reproducibility [27,28] and Shin et al. [29] obtained a very high test–retest reliability, with an intraclass correlation coefficient (ICC) of 0.97. A minimum clinically important difference (MCID) of 1.3 points has been established for patients with neck pain [30]. At each evaluation, the patient’s pain intensity and post-needling soreness in the treatment location were measured. Participants were asked to specifically rate post-needle pain separately from the original myofascial pain in the neck [31]. The measurement of the pain produced during the application of the technique was performed immediately after the intervention.

#### 2.4.2. Neck Disability

The Northwick Park Pain Questionnaire (NPQ) is a self-administered questionnaire for measuring neck disability, which was developed by Gonzalez, T. et al. [32], validated in Spanish and includes 9 sections on daily activities that may be affected by neck pain. Each section contains 1 question and 5 possible answers, which are scored on a scale of 0 to 4, where 4 represents the greatest disability. The score of all responses is summed and converted to a percentage.

This version is a feasible, reliable and valid tool whose test–retest reliability on the total score measured by the ICC is 0.63 [32]. Moreover, it is an instrument with sensitivity to short- and long-term changes [32,33] and the MCID has been set at a 25% reduction in score from baseline [34].

#### 2.4.3. Pressure Pain Threshold

PPT was measured with an analogic pressure algometer (WAGNER Force Dial TM FDK/FDN Series Mechanical Force Gage, Greenwich, CT, USA). The pressure gauge was applied perpendicular to the surface of the active MTrP and was gradually increased at a uniform rate of approximately 1 kg/s. Patients were asked to alert the investigator when they started to feel any discomfort or pain, at which point the pressure was paused and the instrument was immediately withdrawn to read the PPT. Changes in PPT are considered clinically significant when the increase is >15%, compared to baseline [35]. The algometer has good validity and reproducibility [36,37,38]. Several studies [39,40,41] have documented the high intraexaminer and interexaminer reliability of algometer measurements (ICC of 0.80 to 0.97) for PPT assessment in patients with myofascial pain. The mean of three measurements was calculated for each active MTrP in every evaluation.

#### 2.4.4. Cervical Range of Motion

A universal goniometer was used for the measurement, which was placed on the top of the patient’s head. The participants were seated and asked to move their head as much as possible by performing a maximum active ipsilateral rotation towards the affected side. A minimum detectable change of 5° is required for CROM in subjects with neck pain [42]. The universal analogic goniometer has been shown to have good validity for measuring CROM [31] as well as reproducibility [43]. Shamsi et al. [44] gave it an ICC greater than 0.70, in a range from 0.92 to 0.99, which confers high reliability. The mean of three measurements was calculated in each evaluation.

### 2.5. Treatment Allocation

Equal numbers of participants were randomly allocated using the GraphPad statistical and epidemiological analysis software system (GraphPad Software, Inc., La Jolla, CA, USA) to each group: 27 subjects (8 male and 19 female, mean age 39.4 years, body mass index (25.08 kg/m^2^)) were assigned to the DDN group and 27 subjects (8 male and 19 female, mean age 38.2 years, BMI 24.5 kg/m^2^) were assigned to the PE group. 

The study was performed following the COVID-19 safety protocol established by the Community of Madrid (Order 1244/2021, http://www.madrid.org/wleg_pub/secure/normativas/contenidoNormativa.jsf?opcion=VerHtml&nmnorma=12300#no-back-button, accessed on 1 October 2020) for healthcare centers, both in taking measurements and in carrying out interventions. This protocol included the obligatory use of masks and hydroalcoholic gel for patients and physiotherapists, and permanent ventilation and disinfection of the room as well as all of the equipment and materials used with each patient.

The two protocols consisted of a single treatment session and a 14-day follow-up period. Before treatment, VNPS for pain intensity and post-needling soreness, NPQ, CROM and PPT were measured. Afterwards, the assigned intervention was performed and the same assessments were conducted, adding a further VNPS for pain produced during treatment. At 72 h and 14 days, the same assessments were performed as in the pre-intervention phase.

The treatments were performed in the two active MTrPs, on the right or left side, marked in the previous assessment. Both protocols were ultrasound guided, with a portable LOGIQ e BT11^®^ (Enraf Nonius Ibérica S.A., Madrid, Spain) and a high frequency 12L-RS, 5–13 MHz probe. The patients were placed in prone position and the physical therapist performed the following protocol [16]: disinfection of the area with antiseptic solution, application of gloves and probe covers; ultrasound localization of the levator scapulae, performing a longitudinal view of the muscle and considering the superior angle of the scapula as a reference; corresponding safety measures: doppler and measurement of the distance to the point of intervention to choose the correct length of the needle (0.30 mm × 30 or 0.30 mm × 40) (Agu-punt^®^, Madrid, Spain), with a guide tube in the DDN group; and then, needle insertion in the active MTrP until LTR is obtained, which Hong [10] considers a necessary condition for the technique to be effective, and a recent review [15] showed results suggesting an immediate effect on pain intensity when LTRs are obtained. 

### 2.6. Intervention Group: Deep Dry Needling (DDN)

After the first LTR, the “Hong’s [10] fast-in and fast-out” technique is applied. This protocol prolongs the treatment until the LTRs are depleted or the patient’s tolerance is exhausted. In our study, to avoid possible biases, we unified the protocol and according to Fernández-Carnero et al. [45], who did not obtain significant differences in the results according to the dosage: 4, 6 or >6 LTR, a maximum of 4 LTRs were performed in each intervention.

### 2.7. Experimental Group: Percutaneous Electrolysis (PE)

To carry out this treatment, a Physio Invasiva^®^ equipment (Enraf Nonius Ibérica S.A., Madrid, Spain) was employed. Once the first LTR was obtained and following the protocol described by Valera and Minaya [16], the physical therapist activated the galvanic current, and with slow inputs and outputs, performed 3–5 applications of 5 s at an intensity of 1.5 mA.

At the end of the process, in both groups, the needle was removed, the point was compressed for 30 s and the same procedure was repeated in the second active MTrP.

### 2.8. Statistical Analysis

Statistical analyses were performed using SPSS software IBM SPSS Statistics (version 23.0, IBM Corp., Armonk, NY, USA). For quantitative data, mean, standard deviation (SD) and lower and upper limits of the 95% confidence interval (CI), as well as median were calculated, and normality analysis was performed using the Kolmogorov–Smirnov test for sample sizes >50, where the data were considered normally distributed if *p* > 0.05. In addition, the independent Student’s *t*-test for parametric data for the variables that were adjusted to normality (*p* > 0.05) or the U Mann–Whitney nonparametric test for the variables that did not conform to normality (*p* < 0.05) were performed to assess any systematic differences between groups. For these analyses, a *p* value < 0.05 with 95% CI was considered for statistically significant differences. Furthermore, the effect size for outcome measurement differences between both groups was determined by Cohen’s d using the formula d = 2t/√gdl, and interpreted as very small if d was lower than 0.20, small if d varied from 0.20 to 0.49, medium if d varied from 0.50 to 0.79 and large if d was equal of higher than 0.80 [46].

## 3. Results

**Figure 1:**
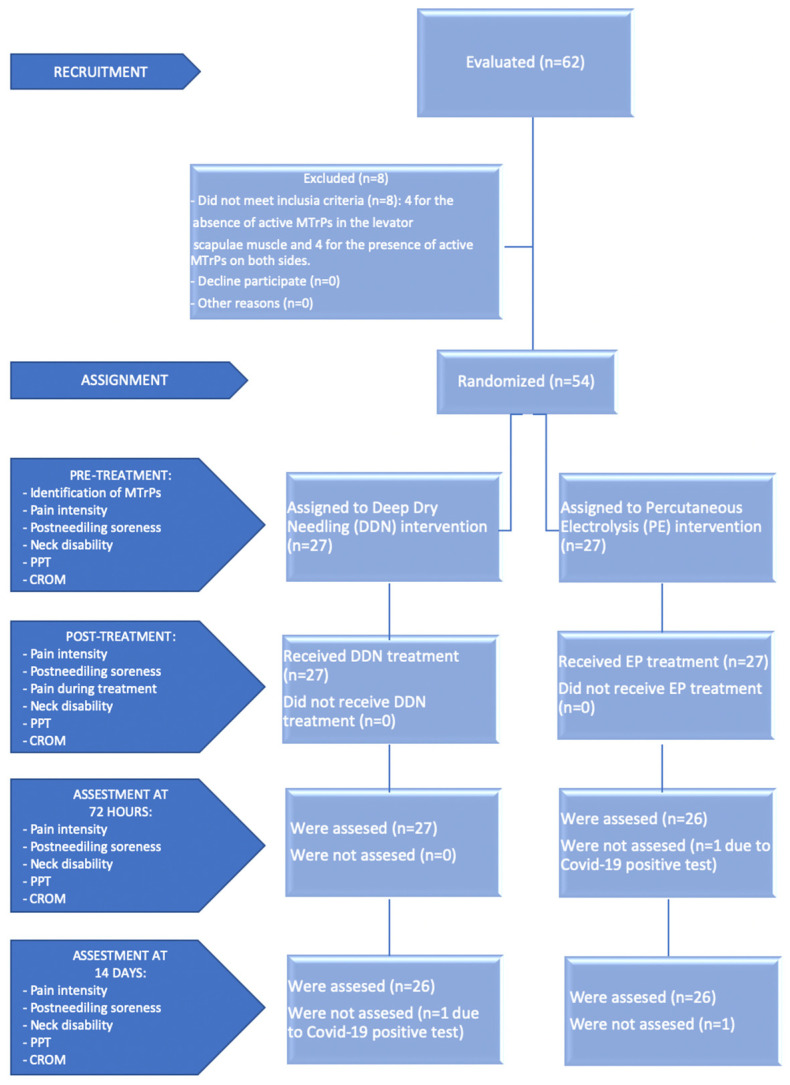
Flow diagram of patients throughout the course of the study. Abbreviations: PPT, pressure pain threshold; CROM, cervical range of motion.

Sixty-two patients were initially recruited to participate in the study. Of all participants, 8 patients were excluded because they did not meet the inclusion criteria. In addition, there were two losses during the study for reasons related to Coronavirus disease (COVID-19). Therefore, there were finally 52 participants who completed it, 26 in each group (Figure 1). None of the subjects presented any adverse effects. 

### 3.1. Sociodemographic Characteristic by Treatment Groups

The demographic data for the studied sample were divided by type of treatment. The intervention group received DDN, and the experimental group received PE. Age, weight, height and BMI have been compared and no statistically significant differences were found between groups since *p* > 0.05 in all cases (Table 1). These results are in line with the randomization of the sample when selecting for one group or another.

### 3.2. Pain Intensity

Table 2 shows that there are no statistically significant differences (*p* > 0.05) at VNPS for pain intensity between groups, before and immediately after treatment, at 72 h and at 14 days (*p* > 0.05; mean differences −0.08–0.23; *d* = 0.02–0.10). Cleland et al. [30] reported that the minimal detectable change (MDC) and minimal clinically important difference (MCID) were 1.3 and 2.1 points, respectively, in patients with mechanical neck pain, so the results in both treatments were clinically relevant at 72 h and 14 days.

### 3.3. Post-Needling Soreness

Table 2 shows that there are no statistically significant differences (*p* > 0.05) at VNPS for post-needling soreness between groups, before and after treatment (*p *> 0.05; mean differences = 0.00–0.46; *d* = 0.00–0.37). Immediately after treatment, the score in the VNPS scale were similar in both groups. At 72 h, in the PE group the score was 0, while in the DDN group, the score was 0.8. At 14 days, post-needling soreness had disappeared in both groups.

### 3.4. Pain Intensity during Treatment

Table 2 shows that there are statistically significant differences (*p* < 0.05) at VNPS for pain intensity during intervention, showing a lower score in patients who received DDN (*p *= 0.02; mean difference = 2; *d* = 0.89).

### 3.5. Neck Disability

Table 2 shows that there are no statistically significant differences before treatment between groups (*p *> 0.05; mean difference = 5.90; *d* = 0.61), but immediately after treatment there are significant differences (*p* < 0.05): patients who received DDN showed a lower percentage of disability (*p *= 0.047; mean difference = 6.1; *d* = 0.63). The relevance of the NPQ improvements immediately after treatment is limited because they did not reach the MCID [34], but at 72 h and at 14 days, clinically relevant results were shown in both groups (*p *> 0.05; mean differences = 0.4 y 1.7; *d* = 0.04 y 0.15 respectively).

### 3.6. Pain Pressure Threshold

Table 3 shows that there are no statistically significant differences (*p* > 0.05) between groups, on PPT at central MTrP (*p *> 0.05; mean differences = 0.02–0.06; *d* = 0.03–0.08) and insertional MTrP (*p *> 0.05; mean differences = 0.08–0.15; *d* = 0.11–0.21), before and after treatment. In addition, the relevance of the PPT improvements obtained in both groups is limited because they did not reach the MDC, 0.45–1.13 kg/cm^2^, [41] for neck pain.

### 3.7. Cervical Range of Motion

Table 3 shows that there are no statistically significant differences before treatment between groups (*p *> 0.05; mean difference = 3.27; *d* = 0.49), but immediately after treatment (*p *= 0.043; mean difference = 3.98; *d* = 0.71) and at 72 h (*p *= 0.045; mean difference = 2.95; *d* = 0.60), there are significant differences (*p* < 0.05), showing a greater ipsilateral maximum active rotation movement for patients who received PE in both measurements. The relevance of the CROM improvements obtained in the present study is limited because they did not reach the MDC for the CROM rotation [42].

## 4. Discussion

Contrary to what we hypothesized, we found no statistically significant differences between groups in terms of pain intensity. To the best of our knowledge, we have not found any studies comparing both techniques in cervical musculature, but in the study conducted by Valera-Calero et al. [47] on the rectus femoris muscle, there was no difference between both treatments with respect to pain intensity either. During the follow-up period, we did not observe these greater regenerative effects attributed to PE [16], given that at 14 days, there were no differences between groups in any of the variables measured. In contrast to our findings, Valera-Calero et al. [47] observed significant improvements with respect to PPT at 7 days in the PE group, but no changes were found in the DDN group. Moreover, in contrast to Valera and Minaya [16], there were no statistically significant differences in post-needling soreness between the two groups. This indicates that in our study, galvanic current did not influence galvanic current in terms of greater or lesser pain at the puncture site, nor did the number of LTRs, since the number of LTRs was not established in the EP protocol. Perhaps these effects attributed to PE will become evident with a greater number of interventions and/or with longer follow-up. Likewise, the fact that the improvements obtained immediately after treatment and at 72 h in CROM and NQP were not sustained over time may have to do with the fact that a greater number of interventions are necessary to maintain the potential benefits. Where the galvanic current seems to have a greater impact than the mechanical effect of the needle itself is on the pain provoked during the intervention, as reflected in our study, given that the VNPS scale showed a significantly higher score for PE versus DDN. This is contrary to Valera-Calero et al. [47], whose results showed that participants who received PE experienced less pain during treatment than the DDN group.

If we analyze similar studies, but with a longer follow-up period, the study by López-Martos et al. [17] on the lateral pterygoid muscle found significant differences at day 28 and day 42, with a greater reduction in VAS scores in the PE group in pain intensity. No statistically significant differences were found between groups in tolerance to treatment, which is contrary to our findings. With respect to range of motion, the increase was greater in the PE group at all times; however, in our study, there were significant differences in favor of the DDN group, but at 14 days, the results were equalized. Finally, with respect to disability, the PE group scores were higher than the DDN group on day 70; however, in our study, there were significant differences in favor of the PE group after treatment, but the results were equalized in the follow-up. Additionally, in the recent research conducted by Al-Boloushi et al. [18] in patients suffering from plantar heel pain caused by MTrPs, at VAS scores, there were differences between groups after 4 weeks of treatment, in favor of the DDN group; however, this difference was not maintained over the time period studies. In our case, no statistically significant differences were found between groups. With respect to disability, only at 52 weeks were differences between groups in favor for the PE group. We cannot draw firm conclusions from these two long-term studies [17,18] since, in our investigation, there was a single intervention and the evaluations were performed in the short term, whereas in their case, several interventions were performed and the first post-treatment evaluation in both studies was at 28 days. 

In view of the results, the mechanical effect of the needle was more important than the effect of the galvanic current. Perhaps, as we pointed out in our study, a greater number of sessions and longer follow-up time are required to obtain more evident differences between the two techniques [17,18]. We thought that the local inflammatory reaction generated by the galvanic current could be a further benefit of the technique, with a greater rupture of the motor end plates of the actin–myosin complexes responsible for the perpetuation of pain, according to prior study suggestions [16,47].

## 5. Limitations

We found an important limitation caused by the Coronavirus disease, which caused difficulties in finding the sample for the study, losses during the evaluations as well as the requirement for incorporating special security measures. In addition to the scarcity of scientific literature on PE, and more specifically, of studies comparing both interventions, DDN and PE, as well as the short follow-up time and the single treatment session could have contributed to the absence of differences between groups in some outcomes. Indeed, future analysis should include the efficacy of each technique separately. It provides new insight into the therapeutic approach’s effectiveness, especially in myofascial pain, which still needs to be better understood. In addition, the lack of a control group that did not receive treatment may be a limitation, as there may be patients who improved naturally over the days. Moreover, as in this study the interventions were ultrasound-guided, this could have led to higher positive patient expectations. Despite our sample size calculation justifying the used sample in our study, a prior systematic review and meta-analysis [8] claimed that larger sample size studies should be carried out as this could substantially change the prevalence of MTrPs. Another limitation that we found in the study is the comparison of the dosage of LTRs, given that it was set at four in the DDN group, but in the PE group, a standardized protocol [16] was followed, regardless of the number of LTRs.

## 6. Conclusions

The protocol [16] used for the PE and the four LTR protocol used in the DDN appear to have similar short-term effects. PE proved to be a more painful treatment than DDN. The results of the studies with a long follow-up period suggest that a greater number of interventions and a longer follow-up time are necessary to observe greater differences between the two. They also show a tendency in the group that received PE to produce longer lasting effects compared to DDN. No adverse effects were observed in either treatment.

## Figures and Tables

**Table 1:** Descriptive data of the participants total population, and dry needling and percutaneous electrolysis groups.

	Total Group N = 52	Dry Needling Group n = 26	Percutaneus Electrolysis Group n = 26	
Descriptive Data	Mean ± SD (95% CI)	Mean ± SD (95% CI)	Mean ± SD (95% CI)	*p* Value
Age (years)	38.77 ± 9.39 (36.15–41.38)	39.35 ± 9.85 (35.37–43.33)	38,19 ± 9.06 (34.53–41.85)	0.700 *
Weight (kg)	69.55 ± 14.37 (65.55–73.55)	69.63 ± 15.48 (63.38–75.89)	69.47 ± 13.47 (64.03–74.90)	0.805 *
Height (m)	1.67 ± 0.09 (1.65–1.70)	1.66 ± 0.10 (1.62–1.70)	1.68 ± 0.09 (1.65–1.72)	0.196 *
BMI (Kg/m^2^)	24.79 ± 4.37 (23.58–26.01)	25.08 ± 4.62 (23.22–26.95)	24.50 ± 4.17 (22.82–26.19)	0.891 *

Abbreviations: BMI, body mass index; Kg, kilograms; M, meters; SD, standard deviation; CI, confidence interval and * U Mann–Whitney test for independent samples were applied. In all analyses, *p* < 0.05 (with a 95% confidence interval) was considered statistically significant.

**Table 2:** Outcome data for VNPS for pain intensity, VPNS for post-needling soreness, VPNS for pain during treatment and NPQ.

	Dry Needling Group N = 26			Percutaneus Electrolysis Group N = 26			
Variable	Mean ± SD (95% CI)	Median (IR)	*p* Value (Kolmogorov–Smirnov)	Mean ± SD (95% CI)	Median (IR)	*p* Value (Kolmogorov–Smirnov)	*p* Value
VNPS neck pain intensity before treatment	6.80 ± 1.13 (6.35–7.26)	6.50 (6.00–7.00)	0.001	6.77 ± 1.03 (6.35–7.19)	7.00 (6.00–7.00)	0.014	0.907 **
VNPS neck pain intensity after treatment	5.23 ± 1.97 (4.44–6.02)	5.00 (4.55–6.00)	0.250	5.04 ± 1.59 (4.40–5.68)	5.00 (4.00–6.00)	0.196	0.772 *
VNPS neck pain intensity 72 h after treatment	3.27 ± 1.87 (2.52–4.02)	3.00 (2.55–4.00)	0.355	3.35 ± 2.10 (2.50–4.19)	3.00 (2.00–4.00)	0.198	0.940 *
VNPS neck pain intensity 14 days after treatment	3.00 ± 2.04 (2.18–3.82)	3.00 (2.00–4.00)	0.164	2.77 ± 2.29 (1.85–3.69)	2.50 (1.00–4.00)	0.042	0.650 **
VNPS post-needling soreness before treatment	0.00 ± 0.00 (0.00–0.00)	0.00 (0.00–0.00)	NA	0.00 ± 0.00 (0.00–0.00)	0.00 (0.00–0.00)	NA	NA
VNPS post-needling soreness after treatment	4.31 ± 2.20 (3.42–5.20)	4.00 (3.00–6.00)	0.178	4.42 ± 2.77 (3.30–5.54)	4.50 (2.00–7.00)	0.009	0.868 **
VNPS post-needling soreness 72 h after treatment	0.88 ± 1.24 (0.38–1.39)	0.00 (0.00–2.00)	<0.0001	0.42 ± 1.27 (−0.09–0.94)	0.00 (0.00–0.00)	<0.0001	0.061 **
VNPS post-needling soreness 14 days after treatment	0.00 ± 0.00 (0.00–0.00)	0.00 (0.00–0.00)	NA	0.00 ± 0.00 (0.00–0.00)	0.00 (0.00–0.00)	NA	NA
VNPS pain during treatment	4.54 ± 2.21 (3.64–5.43)	4.00 (3.00–6.00)	0.3883	6.54 ± 2.27 (5.62–7.45)	7.00 (5.00–8.00)	0.1691	0.002 *
NPQ before treatment	25.9 ± 8 (22.6–29.2)	25.0 (22.2–27.8)	0.1014	31.8 ± 11 (27.5–36.1)	32.3 (23.7–37.4)	0.1126	0.059 *
NPQ after treatment	23.9 ± 8 (20.6–27.2)	23.6 (19.4–26.3)	0.6283	30 ± 11 (25.6–34.5)	29.4 (22.3–36.1)	0.1988	0.047 *
NPQ 72 h after treatment	17.1 ± 9 (13.3–20.9)	16.7 (11.1–22.2)	0.5637	17.5 ± 12 (12.7–22.2)	15.3 (11.9–23.3)	0.3994	0.978 *
NPQ 14 days after treatment	14 ± 11 (9.6–18.4)	13.9 (5.6–16.7)	0.1400	15.7 ± 12 (11.1–20.4)	13.9 (8.3–22.2)	0.1947	0.639 *

Abbreviations: CROM, cervical range of movement; PPT, pain pressure threshold; MTrP, myofascial trigger point; VNPS, Visual Numeric Pain Scale; NPQ, Northwick Park Neck Pain Questionnaire; SD, standard deviation; CI, confidence interval; IR, interquartile range; NA, not applicable; * *p* value from Student’s *t*-test; ** *p* value from U Mann–Whitney. In all analyses, *p* < 0.05 (with a 95% confidence interval) was considered statistically significant.

**Table 3:** Outcome data for PPT on the central MTrP, for PPT on the insertional MTrP and for CROM.

	Dry Needling Group N = 26			Percutaneus Electrolysis Group N = 26			
Variable	Mean ± SD (95% CI)	Median (IR)	*p* Value (Kolmogorov–Smirnov)	Mean ± SD (95% CI)	Median (IR)	*p* Value (Kolmogorov–Smirnov)	*p* Value
PPT on the central MTrP before treatment	2.52 ± 0.70 (2.24–2.80)	2.53 (2.15–3.02)	0.198	2.56 ± 0.75 (2.25–2.86)	2.52 (1.99–2.97)	0.129	0.934 *
PPT on the central MTrP after treatment	2.50 ± 0.71 (2.21–2.78)	2.48 (2–3.02)	0.257	2.56 ± 0.77 (2.25–2.87)	2.52 (2.07–3.05)	0.898	0.700 *
PPT on the central MTrP 72 h after treatment	2.66 ± 0.8 (2.34–2.99)	2.58 (2.09–3.02)	0.130	2.72 ± 0.84 (2.38–3.06)	2.60 (2.23–3.01)	0.683	0.762 *
PPT on the central MTrP 14 days after treatment	2.74 ± 0.81 (2.41–3.06)	2.82 (2.19–3.25)	0.261	2.72 ± 0.77 (2.41–3.03)	2.78 (2.15–3.14)	0.758	0.941 *
PPT on the insertional MTrP before treatment	2.44 ± 0.73 (2.14–2.73)	2.33 (1.89–2.98)	0.074	2.59 ± 0.69 (2.31–2.87)	2.58 (2.12–2.86)	0.292	0.341 *
PPT on the insertional MTrP after treatment	2.36 ± 0.64 (2.11–2.62)	2.42 (1.82–2.82)	0.387	2.44 ± 0.76 (2.13–2.74)	2.38 (2.11–2.58)	0.082	0.978 *
PPT on the insertional MTrP 72 h after treatment	2.51 ± 0.66 (2.25–2.78)	2.43 (2.13–2.68)	0.153	2.60 ± 0.81 (2.27–2.92)	2.52 (2.09–2.80)	0.039	0.790 **
PPT on the insertional MTrP 14 days after treatment	2.48 ± 0.69 (2.20–2.76)	2.38 (2.20–2.65)	0.461	2.56 ± 0.60 (2.32–2.80)	2.58 (2.25–2.95)	0.632	0.533 *
CROM of ipsilateral rotation before treatment	68.79 ± 8.50 (65.36–72.23)	71 (66.07–73.63)	0.002	72.06 ± 4.04 (70.43–73.70)	72.33 (70.37–72.97)	0.402	0.368 **
CROM of ipsilateral rotation after treatment	73.90 ± 7.35 (70.93–76.86)	76 (74–78)	0.000	77.88 ± 2.89 (76.72–79.05)	78 (76–79.48)	0.683	0.043 **
CROM of ipsilateral rotation 72 h after treatment	74.54 ± 6 (72.12–76.96)	74.67 (72.67–77.63)	0.183	77.49 ± 3.58 (76.04–78.93)	78.67 (75.33–79.33)	0.181	0.045 *
CROM of ipsilateral rotation 14 days after treatment	74.13 ± 4.97 (72.12–76.14)	74.67 (72.37–76.60)	0.071	75.13 ± 3.84 (73.58–76.68)	75 (73.03–77.27)	0.708	0.653 *

Abbreviations: CROM, cervical range of movement; PPT, pain pressure threshold; MTrP, myofascial trigger point; VNPS, Visual Numeric Pain Scale; NPQ, Northwick Park Neck Pain Questionnaire; SD, standard deviation; CI, confidence interval; IR, interquartile range; NA, not applicable; * *p* value from Student’s *t*-test; ** *p* value from U Mann–Whitney. In all analyses, *p* < 0.05 (with a 95% confidence interval) was considered statistically significant.

## Data Availability

The data will be available under request.

## References

[1] Borghouts J.A.J., Koes B.W., Bouter L.M. (1998). The clinical course and prognostic factors of non-specific neck pain: A systematic review. Pain.

[2] Schellingerhout J.M., Verhagen A.P., Heymans M.W., Pool J.J.M., Vonk F., Koes B.W., de Vet H.C.W. (2008). Which subgroups of patients with non-specific neck pain are more likely to benefit from spinal manipulation therapy, physiotherapy, or usual care?. Pain.

[3] Vonk F., Verhagen A.P., Twisk J.W., Köke A.J.A., Luiten M.W.C.T., Koes B.W. (2009). Effectiveness of a behaviour graded activity program versus conventional exercise for chronic neck pain patients. Eur. J. Pain.

[4] Cohen S.P. (2015). Epidemiology, diagnosis, and treatment of neck pain. Mayo Clin. Proc..

[5] Instituto Nacional de Estadística (INE), Ministerio de Sanidad (MS) (2020). Encuesta Europea de Salud en España (EESE 2020). https://www.ine.es/dynt3/inebase/es/index.htm?type=pcaxis&path=/t15/p420/a2019/p01/&file=pcaxis.

[6] Cerezo-Téllez E., Torres-Lacomba M., Mayoral-del Moral O., Sánchez-Sánchez B., Dommerholt J., Gutiérrez-Ortega C. (2016). Prevalence of myofascial pain syndrome in chronic non-specific neck pain: A population- based cross-sectional descriptive study. Pain Med..

[7] Gallego-Sendarrubias G.M., Rodríguez-Sanz D., Calvo-Lobo C., Martín J.L. (2020). Efficacy of dry needling as an adjunct to manual therapy for patients with chronic mechanical neck pain: A randomised clinical trial. Acupunct. Med..

[8] Chiarotto A., Clijsen R., Fernandez-De-Las-Penas C., Barbero M. (2016). Prevalence of Myofascial Trigger Points in Spinal Disorders: A Systematic Review and Meta-Analysis. Arch. Phys. Med. Rehabil..

[9] Fernaández-de-las-Peñas C., Cleland J., Huijbregts P. (2011). Neck and Arm Pain Syndromes: Evidence-Informed Screening, Diagnosis and Management.

[10] Hong C.Z. (1994). Lidocaine injection versus dry needling to myofascial trigger point: The importance of the local twitch response. Am. J. Phys. Med. Rehabil..

[11] Abat F., Diesel W., Gelber P., Polidori F., Monllau J. (2014). Effectiveness of the Intratissue Percutaneous Electrolysis (EPI^®^) technique and isoinertial eccentric exercise in the treatment of patellar tendinopathy at two years follow-up. Muscle Ligaments Tendons J..

[12] Abat F., Gelber P.E., Polidori F., Monllau J.C., Sanchez-Ibañez J.M. (2015). Clinical results after ultrasound-guided intratissue percutaneous electrolysis (EPI^®^) and eccentric exercise in the treatment of patellar tendinopathy. Knee Surgery, Sport. Traumatol. Arthrosc..

[13] Gattie E., Cleland J.A., Snodgrass S. (2017). The effectiveness of trigger point dry needling for musculoskeletal conditions by physical therapists: A systematic review and meta-analysis. J. Orthop. Sport. Phys. Ther..

[14] Sánchez-Infante J., Navarro-Santana M.J., Bravo-Sanchez A., Jimenez-Diaz F., Abian-Vicen J. (2021). Is dry needling applied by physical therapists effective for pain in musculoskeletal conditions? A systematic review and meta-analysis. Phys. Ther..

[15] Fernández-de-Las-Peñas C., Plaza-Manzano G., Sanchez-Infante J., Gómez-Chiguano G.F., Cleland J.A., Arias-Buría J.L., Navarro-Santana M.J. (2022). The importance of the local twitch response during needling interventions in spinal pain associated with myofascial trigger points: A systematic review and meta-analysis. Acupunct. Med..

[16] Valera F., Minaya M. (2017). Fisioterapia Invasiva.

[17] Lopez-Martos R., Gonzalez-Perez L.M., Ruiz-Canela-Mendez P., Urresti-Lopez F.J., Gutierrez-Perez J.L., Infante-Cossio P. (2018). Randomized, double-blind study comparing percutaneous electrolysis and dry needling for the management of temporomandibular myofascial pain. Med. Oral Patol. Oral Cir. Bucal.

[18] Al-Boloushi Z., Gómez-Trullén E.M., Arian M., Fernández D., Herrero P., Bellosta-López P. (2020). Comparing two dry needling interventions for plantar heel pain: A randomised controlled trial. BMJ Open.

[19] García Naranjo J., Barroso Rosa S., Loro Ferrer J.F., Limiñana Cañal J.M., Suarez Hernández E. (2017). A novel approach in the treatment of acute whiplash syndrome: Ultrasound-guided needle percutaneous electrolysis. A randomized controlled trial. Orthop. Traumatol. Surg. Res..

[20] Simons Travell J.G., Simons L.S., Travell J.G., David G. (1999). Travell & Simons’ Myofascial Pain and Dysfunction: The Trigger Point Manual.

[21] Fernández-de-las-Peñas C., Alonso-Blanco C., Miangolarra J.C. (2007). Myofascial trigger points in subjects presenting with mechanical neck pain: A blinded, controlled study. Man. Ther..

[22] Muñoz-Muñoz S., Muñoz-García M.T., Alburquerque-Sendín F., Arroyo-Morales M., Fernández-De-Las-Peñas C. (2012). Myofascial trigger points, pain, disability, and sleep quality in individuals with mechanical neck pain. J. Manip. Physiol. Ther..

[23] Travell J., Simons D. (1983). Myofascial Pain and Dysfunction: The Trigger Point Manual.

[24] Fernández-de-las-Peñas C., Dommerholt J. (2018). International consensus on diagnostic criteria and clinical considerations of myofascial trigger points: A delphi study. Pain Med..

[25] Myburgh C., Larsen A.H., Hartvigsen J. (2008). A Systematic, Critical Review of Manual Palpation for Identifying Myofascial Trigger Points: Evidence and Clinical Significance. Arch. Phys. Med. Rehabil..

[26] González V.M., Stewart A., Ritter P.L., Lorig K. (1995). Translation and validation of arthritis outcome measures into spanish. Arthritis Rheum..

[27] Price D.D., McGrath P.A., Rafii A.B.B. (1983). The validation of visual analogue scales as ratio scale measures for chronic and experimental pain. Pain.

[28] Gattie E.R., Cleland J.A., Snodgrass S.J. (2017). Dry Needling for Patients with Neck Pain: Protocol of a Randomized Clinical Trial. JMIR Res. Protoc..

[29] Shin Y.J., Kim W.H., Kim S.G. (2017). Correlations among visual analogue scale, neck disability index, shoulder joint range of motion, and muscle strength in young women with forward head posture. J. Exerc. Rehabil..

[30] Cleland J.A., Childs J.D., Whitman J.M. (2008). Psychometric Properties of the Neck Disability Index and Numeric Pain Rating Scale in Patients with Mechanical Neck Pain. Arch. Phys. Med. Rehabil..

[31] León-Hernández J., Martín-Pintado-Zugasti A., Frutos L., Alguacil-Diego I., de la Llave-Rincón A., Fernandez-Carnero J. (2016). Immediate and short-term effects of the combination of dry needling and percutaneous TENS on post-needling soreness in patients with chronic myofascial neck pain. Braz. J. Phys. Ther..

[32] González T., Balsa A., de Murieta J.S., Zamorano E., González I., Martin-Mola E. (2001). Spanish version of the Northwick Park neck pain questionnaire: Reliability and validity. Clin. Exp. Rheumatol..

[33] Leak A.M., Frank A.O. (1994). The northwick park neck pain questionnaire, devised to measure neck pain and disability. Rheumatology.

[34] Sim J., Jordan K., Lewis M., Hill J., Hay E.M., Dziedzic K. (2006). Sensitivity to change and internal consistency of the Northwick Park neck pain questionnaire and derivation of a minimal clinically important difference. Clin. J. Pain.

[35] Voogt L., de Vries J., Meeus M., Struyf F., Meuffels D., Nijs J. (2015). Analgesic effects of manual therapy in patients with musculoskeletal pain: A systematic review. Man. Ther..

[36] Kinser A.M., Sands W.A., Stone M.H. (2009). Reliability and validity of a pressure algometer. J. Strength Cond. Res..

[37] List T., Helkimo M., Falk G. (1989). Reliability and validity of a pressure threshold meter in recording tenderness in the masseter muscle and the anterior temporalis muscle. Cranio-J. Craniomandib. Pract..

[38] Fischer A.A. (1987). Pressure algometry over normal muscles. Standard values, validity and reproducibility of pressure threshold. Pain.

[39] Park G., Kim C.W., Park S.B., Kim M.J., Jang S.H. (2011). Reliability and Usefulness of the Pressure Pain Threshold Measurement in Patients with Myofascial Pain. Ann. Rehabil. Med..

[40] Chung S.C., Um B.Y., Kim H.S. (1992). Evaluation of pressure pain threshold in head and neck muscles by electronic algometer: Intrarater and interrater reliability. Cranio.

[41] Walton D., Macdermid J., Nielson W., Teasell R., Chiasson M., Brown L. (2011). Reliability, standard error, and minimum detectable change of clinical pressure pain threshold testing in people with and without acute neck pain. J. Orthop. Sports Phys. Ther..

[42] Fletcher J.P., Bandy W.D. (2008). Intrarater Reliability of CROM Measurement of Cervical Spine Active Range of Motion in Persons with and without Neck Pain. J. Orthop. Sport. Phys. Ther..

[43] Farooq M.N., Mohseni Bandpei M.A., Ali M., Khan G.A. (2016). Reliability of the universal goniometer for assessing active cervical range of motion in asymptomatic healthy persons. Pak. J. Med. Sci..

[44] Shamsi M.B., Mirzaei M., Khabiri S.S. (2019). Universal goniometer and electrogoniometer intra-examiner reliability in measuring the knee range of motion during active knee extension test in patients with chronic low back pain with short hamstring muscle. BMC Sport. Sci. Med. Rehabil..

[45] Fernández-Carnero J., Gilarranz-de-Frutos L., León-Hernández J.V., Pecos-Martin D., Alguacil-Diego I., Gallego-Izquierdo T., Martín-Pintado-Zugasti A. (2017). Effectiveness of Different Deep Dry Needling Dosages in the Treatment of Patients with Cervical Myofascial Pain: A pilot RCT. Am. J. Phys. Med. Rehabil..

[46] Cohen J. (2013). Statistical Power Analysis for the Behavioral Sciences.

[47] Valera-Calero J.A., Sánchez-Mayoral-Martín A., Varol U. (2021). Short-term effectiveness of high- and low-intensity percutaneous electrolysis in patients with patellofemoral pain syndrome: A pilot study. World J. Orthop..

